# Enhancing salt stress tolerance in wheat (*Triticum aestivum*) seedlings: insights from trehalose and mannitol

**DOI:** 10.1186/s12870-024-04964-2

**Published:** 2024-05-30

**Authors:** Abdulrahman M. Alhudhaibi, Mervat A. R. Ibrahim, Seham M. S. Abd-Elaziz, Hanaa R. M. Farag, Salwa M. Elsayed, Hemmat A. Ibrahim, ABM Sharif Hossain, Basmah M. Alharbi, Faouzi Haouala, Amr Elkelish, Hany A. M. Srour

**Affiliations:** 1 Department of Biology, College of Science, Imam Mohammad Ibn Saud Islamic University, Riyadh, 11623 Kingdom of Saudi Arabia; 2 Biochemistry Department, Faculty of Agriculture, Ain Shams University, 11241 Shoubra Alkheima, Cairo Egypt; 3 Biology Department, Faculty of Science, University of Tabuk, Tabuk, 71491 Saudi Arabia; 4 Biodiversity Genomics Unit, Faculty of Science, University of Tabuk, Tabuk, 71491 Saudi Arabia; 5 Botany Department, Faculty of Science, Suez Canal University, Ismailia, Egypt

**Keywords:** Trehalose, Mannitol, Wheat (*Triticum estivium*), Salt stress, Antioxidant enzymes

## Abstract

Salinity stress, an ever-present challenge in agriculture and environmental sciences, poses a formidable hurdle for plant growth and productivity in saline-prone regions worldwide. Therefore, this study aimed to explore the effectiveness of trehalose and mannitol induce salt resistance in wheat seedlings. Wheat grains of the commercial variety Sakha 94 were divided into three groups : a group that was pre-soaked in 10 mM trehalose, another group was soaked in 10 mM mannitol, and the last was soaked in distilled water for 1 hour, then the pre soaked grains cultivated in sandy soil, each treatment was divided into two groups, one of which was irrigated with 150 mM NaCl and the other was irrigated with tap water. The results showed that phenols content in wheat seedlings increased and flavonoids reduced due to salt stress. Trehalose and mannitol cause slight increase in total phenols content while total flavonoids were elevated highy in salt-stressed seedlings. Furthermore, Trehalose or mannitol reduced salt-induced lipid peroxidation. Salt stress increases antioxidant enzyme activities of guaiacol peroxidase (G-POX), ascorbate peroxidase (APX), and catalase (CAT) in wheat seedlings, while polyphenol oxidase (PPO) unchanged. Trehalose and mannitol treatments caused an increase in APX, and CAT activities, whereas G-POX not altered but PPO activity were decreased under salt stress conditions. Molecular docking confirmed the interaction of Trehalose or mannitol with peroxidase and ascorbic peroxidase enzymes. Phenyl alanine ammonia layase (PAL) activity was increased in salt-stressed seedlings. We can conclude that pre-soaking of wheat grains in 10 mM trehalose or mannitol improves salinity stress tolerance by enhancing antioxidant defense enzyme and/or phenol biosynthesis, with docking identifying interactions with G-POX, CAT, APX, and PPO.

## Introduction

Salinity stress is one of the major environmental stresses that decrease plant productivity in several areas of the world. Salt stress caused remarkable negative effects on plant metabolism and growth [1, 2]. Wheat is one of the most important strategic crops in Egypt. The global annual production of wheat is about 736 million metric tons regarding FAO 2015. Wheat production is declined continuously due to global climate changes [3].As climate change and rising temperatures lead to increased evaporation of water from the soil, thus salt remains in the soil, which increases soil salinity [4]. Increasing of water and soil salinity led to remarkable decreases in wheat productivity. The salinity problem is solved by removing salts out of the soil through excessive irrigation [5]. To counteract their harmful effects, plants have formed antioxidant enzymatic systems including ascorbate peroxidase (APX), superoxide dismutase (SOD), catalase (CAT), glutathione reductase (GR), peroxidase (POD) and non-enzymatic scavengers like phenolic compounds, sulfur tripeptide (glutathione), vitamin C (L-ascorbic acid), vitamin E and carotenoids [6]. The enzymatic and non enzymatic antioxidants aimed to scavenge the reactive oxygen species such as Hydrogen peroxide, H_2_O_2_, superoxide radicals, O_2_, and hydroxyl radicals (OH.), which causes significant damage of lipids and proteins [7, 8].

Trehalose is an important compatible solute and non-reducing sugar which is formed in several tissues of different organisms, including prokaryotes and eukaryotes. In many plants’ species, Trehalose is not detected, except under specific conditions, some plant species produce accumulate Trehalose in detectable quantities. Many researchers have shown that formation and accumulation of Trehalose can stabilize some proteins and biological membranes in some microorganisms under stress [9, 10]. Also, Trehalose was found to be accumulated in genetic modified plants and induced their tolerance to abiotic stress [11, 12]. The biochemical and molecular mechanisms of the induced resistance by Trehalose are still unclear.

Mannitol is an alcoholic sugar formed and accumulated in many plant species, not including wheat (*Triticum aestivum*). For instance, in celery (*Apium graveolens*), mannitol and sucrose are synthesized in equal amount. Mannitol accumulates when plants are subjected to drought or low water potential [13]. The accumulation of mannitol is regulated through its metabolism [14, 15]. Salinity stress caused significant inhibition to sucrose synthesis pathway and does not affect mannitol biosynthesis pathways in celery plant. Moreover, the utilization rate of mannitol in sink tissues significantly decreases during salt stress due to the down regulation of the NAD^+^-dependent mannitol dehydrogenase, which oxidizes mannitol to Mannose [16, 17]. Tarczynski et al. and Thomas et al. showed that in mannitol accumulating transgenic tobacco (*Nicotiana tabacum*) and Arabidopsis plants, the plants can grow under high salt conditions [18, 19]. Similar to Trehalose, the molecular mechanisms that mannitol can induce plant tolerance to salt stress is not clear. So, the present work investigated the ability of Trehalose and mannitol to improve salinity tolerance of wheat seedlings (*Triticum aestivum* Var. Sakha 94). Also, the effect of pre-soaking of wheat grains in Trehalose and mannitol on the antioxidant defensive enzymes ( peroxidase, ascorbate peroxidase, catalase and polyphenol oxidase), non enzymatic antioxidant (phenols and flavonoids) and others stress markers which accumulated under salt stress.

## Materials and methods

### The treatments and growth conditions

Wheat grains (*Triticum estivum*, L. cv. Sakha 94) were obtained from the agriculture research center, Ministry of Agriculture, Giza, Egypt. Wheat grains were cleaned by using sodium hypochlorite (2%) then washed with tap water. In treatments, the grains were divided into three groups: The first group was treated with water and the other two groups were treated with either Trehalose (10 mM) or mannitol (10mM). The grains were placed between two layers of paper towels, and then water, trehalose or mannitol solution was added to cover all the grains and kept for one hour at 28°C. Then, the treated grains were washed with tap water. Grains of each group were divided into two subgroups: the first one was cultivated in washed sandy soil and irrigated with tap water, while the second subgroup was cultivated in washed sandy soil irrigated with NaCl solution (150 mM). 10 replicates for each treatment were grown in a complete randomized design. After 21 days of regular irrigation,Sixth plants were randomly were collected before sun rise, washed and the leaves were stored at -22°C for biochemical analysis.

### Chemical determinations

#### Reducing sugars, total soluble sugars and Free amino acids

Total soluble sugars and free amino acids were extracted from 0.5 g leaves by ethanol 80% at 70 ℃ for 1 hour and repeated three times according to Ackerson [20]. Reducing sugars were determined in this ethanolic extract by 3,5 dinitrosalicylic reagent, as described by Negrulescu et al. [21] using glucose as standard. Total soluble sugars was determined in the previous extract after acid hydrolysis by HCl 2N at 60 ℃ for 30 min, then neutralized and estimated by 3,5 dinitrosalicylic reagent.

Free amino acids were measured colorimetrically using a ninhydrin solution according to Jayeraman [22] using lysine as standard.

#### Proline

Proline content was measured using ninhydrin reagent, as described by Mendel Friedman 2004 [23],

#### Phenolic compounds and total flavonoids

Phenolic compounds and total flavonoids were extracted by macerated 0.5 g of fresh leaves in 10 ml 80% ethanol for 24 h at 5 ℃ and repeated three times. The collected extracts were completed to 50 ml using 80% ethanol.Total flavonoids content was determined in the previous extract by the aluminum chloride method which described by chang et al. [24] using quercetin as standard. Phenolic compounds content was determined by Folin-Ciocalteu reagent according to the method described by Duca et al. [25] using gallic acid as standard.

#### Lipid peroxidation

Lipid peroxidation was determined by measuring the concentration of malondialdhyde (MDA) in fresh leaves according to the method described by Gérard-Monnier et al. [26].

#### Enzymes assay

##### Preparation of crude enzyme extract

Plant seedling (500 mg) was homogenized in Potassium phosphate buffer (100 mM, pH = 7.0) .Homogenate was centrifuged at 15,000g for 15 min at 4 ℃ and supernatant was used to measure the activities of phenyl alanine ammonia layase (PAL) and the antioxidant enzymes( APX, G-POX,PPO and CAT).

##### Assay of ascorbate peroxidase activity(APX)

Ascorbate peroxidase, APX (E.C 1.11.1.11) activity was determined through measuring the reduction of the absorbance at 290 nm for 1 min using L-ascorbate as standard according to Balestri et al. [27].

##### Assay of guaiacol peroxidase activity(G-POX)

The enzyme crude extract was tested for guaiacol peroxidase, G-POX (E.C. 1.11.1.7) activity according to the methods published by Simões et al. [28]. POD activity was calculated based on the molar extinction coefficient of 26.6 mM^-1^ cm^-1^ for guaiacol at 470 nm and expressed in µmol min^-1^ g FM^-1^.

##### Assay of polyphenoloxidase activity (PPO)

Polyphenol oxidase (PPO) (EC 1.14.18.1) activity was estimated according to Simões et al. [28], method. PPO activity was calculated based on the molar extinction coefficient of 3400 mM^-1^ cm^-1^ for catechol at 420 nm and expressed in µmol min^-1^ g FM^-1^.

##### Assay of catalase activity (CAT)

Using a technique created by Aeby [29], the activity of the catalase, CAT (E.C 1.11.1.6), was assessed. The activity was calculated from extinction coefficient (ε = 40 mM^-1^ cm^-1^) for H_2_O_2_. One unit of enzyme activity was defined as the decomposition of 1 mol of H_2_O_2_ per minute at 240 nm.

##### Assay of phenylalanine ammonia-lyase activity (PAL)

The method outlined by He et al. [30] was used to measure the activity of phenylalanine ammonia-lyase, or PAL (E.C. 4.3.1.5). One unit of enzyme activity was defined as the amount of enzyme that caused an increase in absorbance of 0.01 per hour at 290 nm.

#### Soluble proteins

Soluble Proteins concentration was quantified in the crude enzyme extract by Bradford METHODS according to Philomina et al. [31] using bovine serum albumin as a standard.

### Molecular docking

The molecular interaction study was carried out using autodock4 [32]. Ascorbate peroxidase structure 1oaf [33], peroxidase structure 1sch [34] and polyphenol oxidase structure 1bt1 [35] were obtained from the protein data bank. A 40 × 40 × 58Å grid box with 16.282× 64.422× 24.376 grid point spacing of 0.375 ˚ A, a 60 × 72 × 60Å grid box with 15.935 × 32.615 × 57.859 grid point spacing of 0.375 ˚ A and a 40 × 40 × 40 grid box with 36.211 × 132.950 × 45.627 grid point spacing of 0.375 ˚ A were employed for ascorbate peroxidase, peroxidase and polyphenol oxidase respectively. The ligands structure, Trehalose and mannitol were drawn using PubChem draw structure tools. Autodock 4 software was used to generate several docking poses and to select the pose with the optimum energy score.

### Statistical analysis

The statistical analysis was conducted employing six replicates for every treatment. All collected data represent the mean value derived from six individual samples, with the standard deviation (SD) indicating the variability within the dataset. The acquired outcomes were examined through one-way analysis of variance (ANOVA) employing Costat software (Version 6.303), as per Stern [36]. The determination of the significance of the disparity between treatments was conducted by employing Duncan's multiple range test at a significance level of *P* ≤ 0.05.

## Results

### Protein, free amino acids, proline, total soluble and reducing sugars

**Fig. 1:**
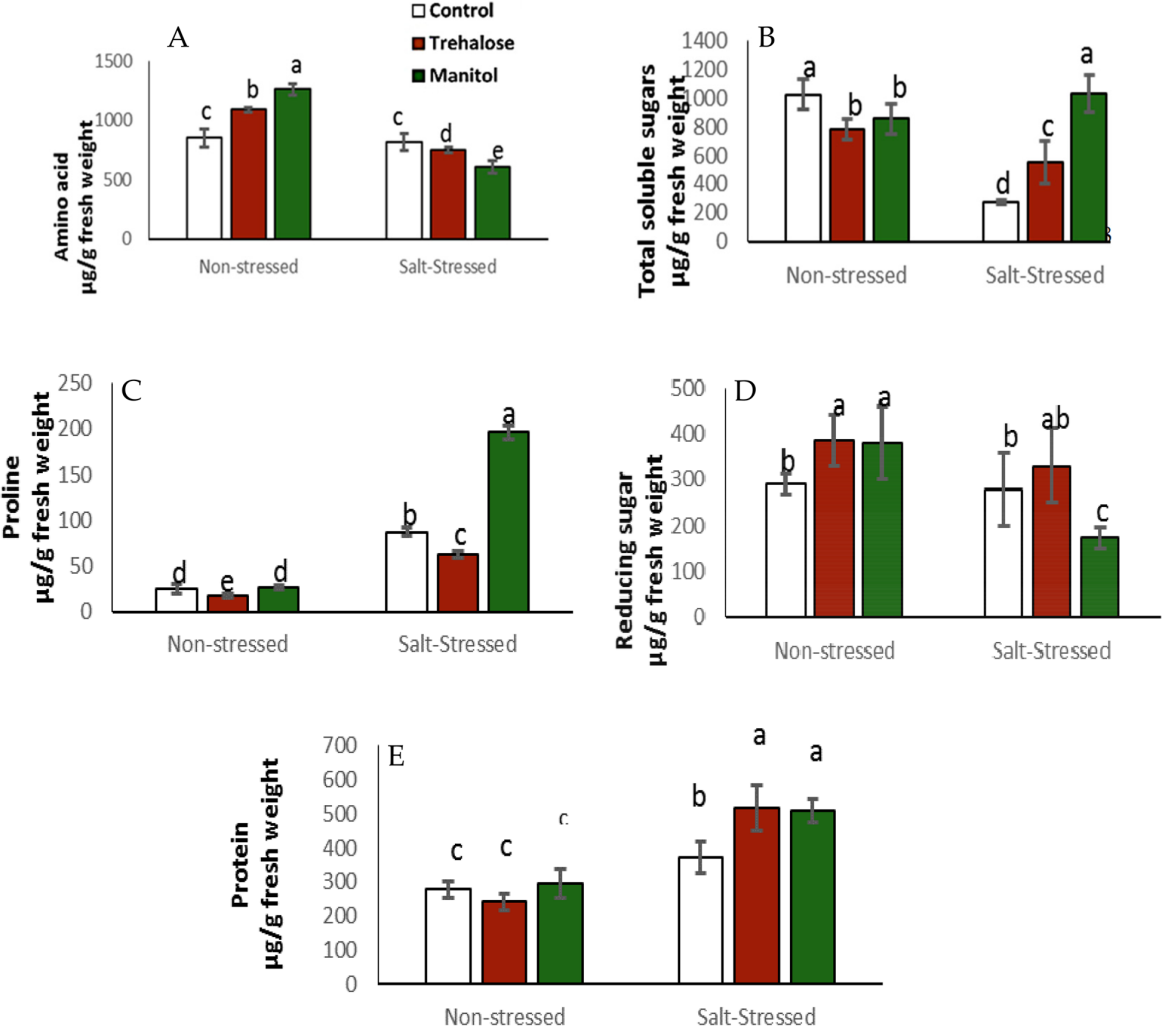
Effect of wheat grain pre-soaking in 10 mM trehalose and mannitol on amino acids, proline, total soluble sugar, reducing sugar and protein content in wheat seedlings grown salty condition

As shown in Fig. 1, it indicates that trehalose and mannitol treatments in non-stressed conditions caused a significant increase in free amino acids contents compared with non-stressed seedlings in the control group. On the other hand, in stressed conditions, both treatments significantly decreased the amino acid content as compared with untreated control. Also, Trehalose and mannitol significantly increased total soluble sugar (TSS) in salt-stressed plants as compared with control (see Fig. 1B). By contrast, the concentration of reducing sugars was significantly reduced in the case of mannitol under salinity stress, while there was no significant difference between Trehalose and control (Fig. 1D). All treatments under salinity stress led to a remarkable increase in proline content compared to non-stressed plants.

### Total flavonoids and phenolic compounds

**Fig. 2:**
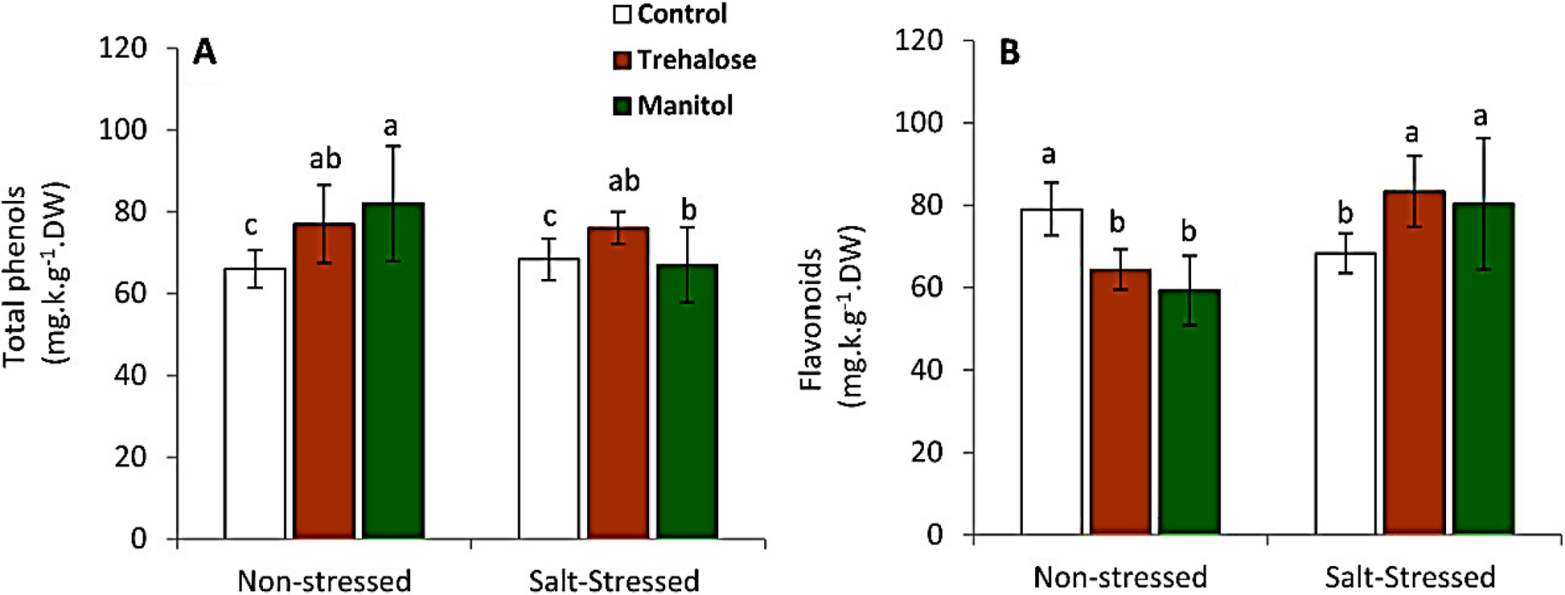
Effect of wheat grain pre-soaking in 10 mM trehalose and mannitol on total phenols and flavonoids contents in wheat seedlings grown under salt stress condition.

Pre soaking of wheat grains in trehalose resulted in a significant rise in seedling content of phenolic compounds compared to soaking grains in mannitol in plants exposed to salt stress. Wheat seedlings treated with Trehalose and mannitol before planting showed significant increases in flavonoids compared to untreated plant under salt stress (Fig. 2). The wheat seedlings treated with Trehalose and mannitol before planting caused a significant increase in phenols of non stressed plants compared to control, as illustrated in (Fig. 2). While, Trehalose and mannitol caused a significant reduction in flavonoid content of non-stressed wheat seedlings compared to the control.

### Lipid peroxidation

**Fig. 3:**
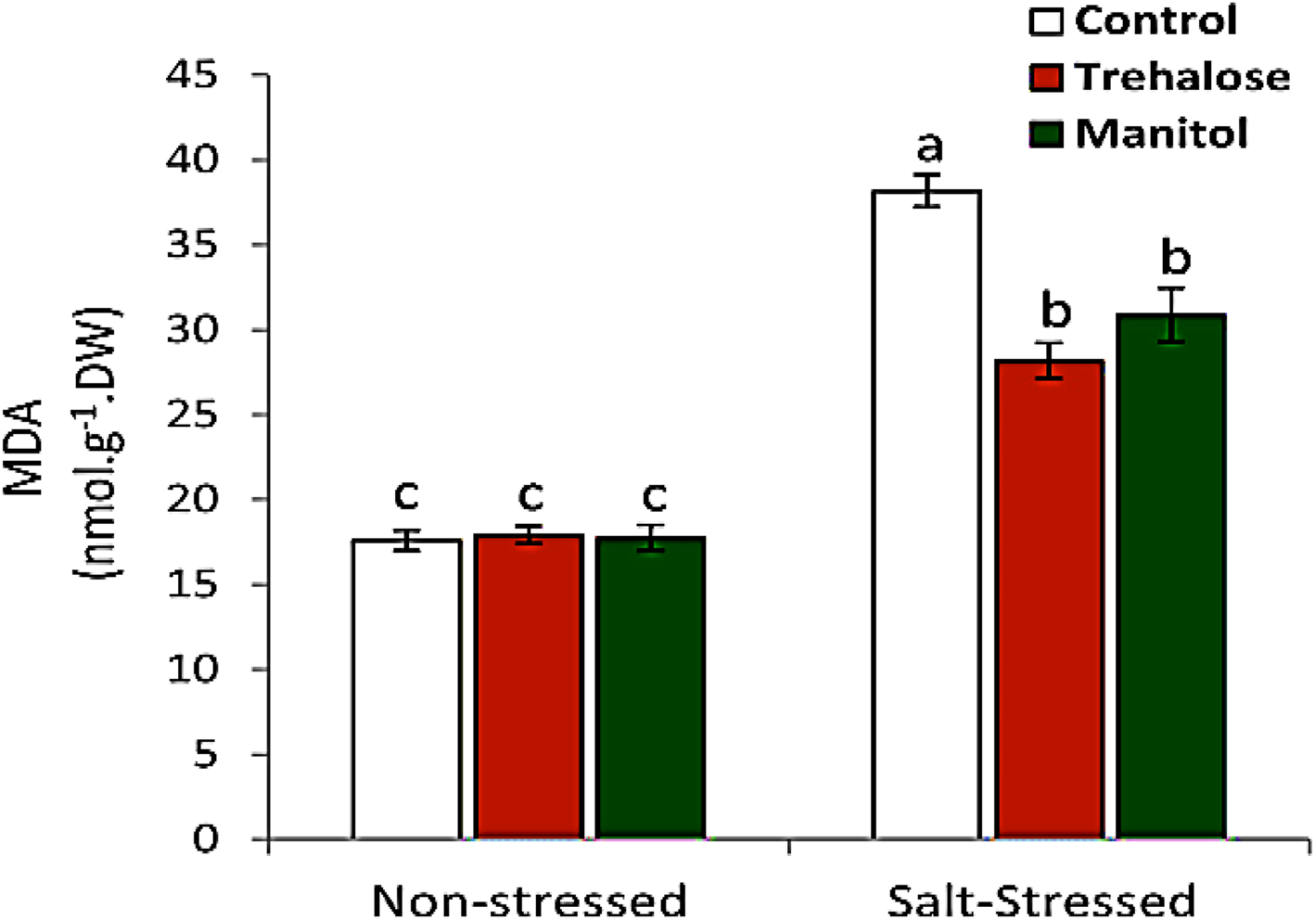
Effect of wheat grain pre-soaking in 10 mM trehalose and mannitol on MDA contents in wheat seedlings grown under salt stress condition

As showed in Fig. 3 showed the level of lipid peroxidation in different treatments. Data clearly showed that salt stress resulted in a remarkable increase in the level of lipid peroxidation whereas, MDA content is duplicated due to salt stress. Pre-soaking of wheat grains in 10 mM Trehalose or 10 mM mannitol led to significant reduction in MDA contents in salt stressed plant and both treatments have no effect on the level of MDA in non-stressed seedlings.

### Antioxidant enzymes

**Fig. 4:**
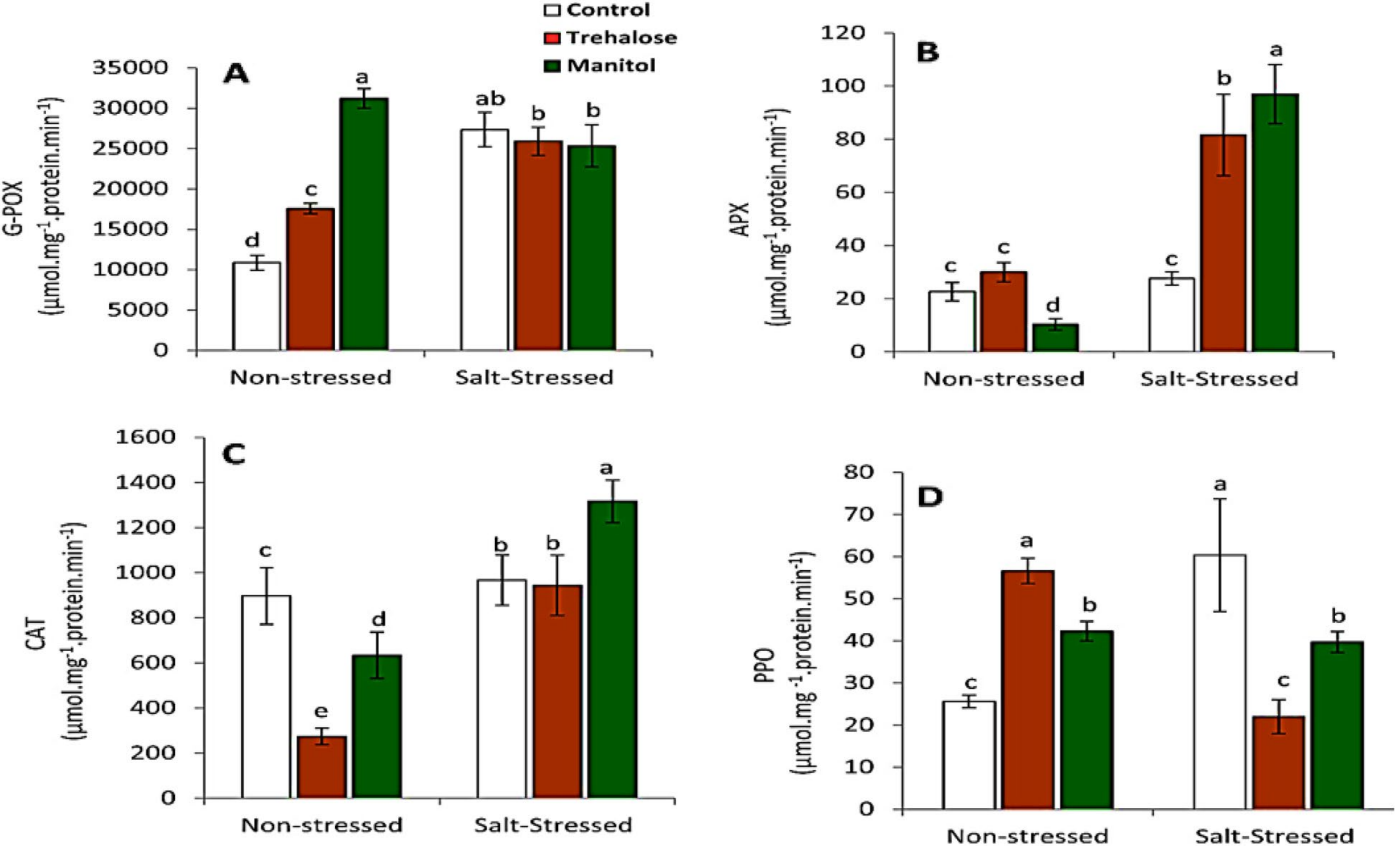
Effect of wheat grain pre-soaking in 10 mM trehalose and mannitol on antioxidant enzymes (**A**) guaiacol peroxidase (G-POX), **B** ascorbate peroxidase (APX), **C** catalase (CAT), and PPO activities in wheat seedlings grown under salt stress condition

Seedlings grown under salinity stress condition showed significant increases in the activity of enzymes namely G-POX, CAT, APX and PPO (Fig. 4). Application of exogenous pretreatments with Trehalose and mannitol seem to promote the activity of G-POX and PPO enzymes compared to control of non-stressed plants, While the same treatments led to significant decrease in the activity of catalase enzymes in non stressed wheat seedlings. Interestingly, application of mannitol led to increase in the activity of CAT and APX **(**Fig. 4B and C*)* in salt stressed plants in comparison with that in non-stressed plants. Moreover, mannitol effectively increased APX, CAT and PPO activities in the presence of NaCl stress compared to Trehalose.

**Fig. 5:**
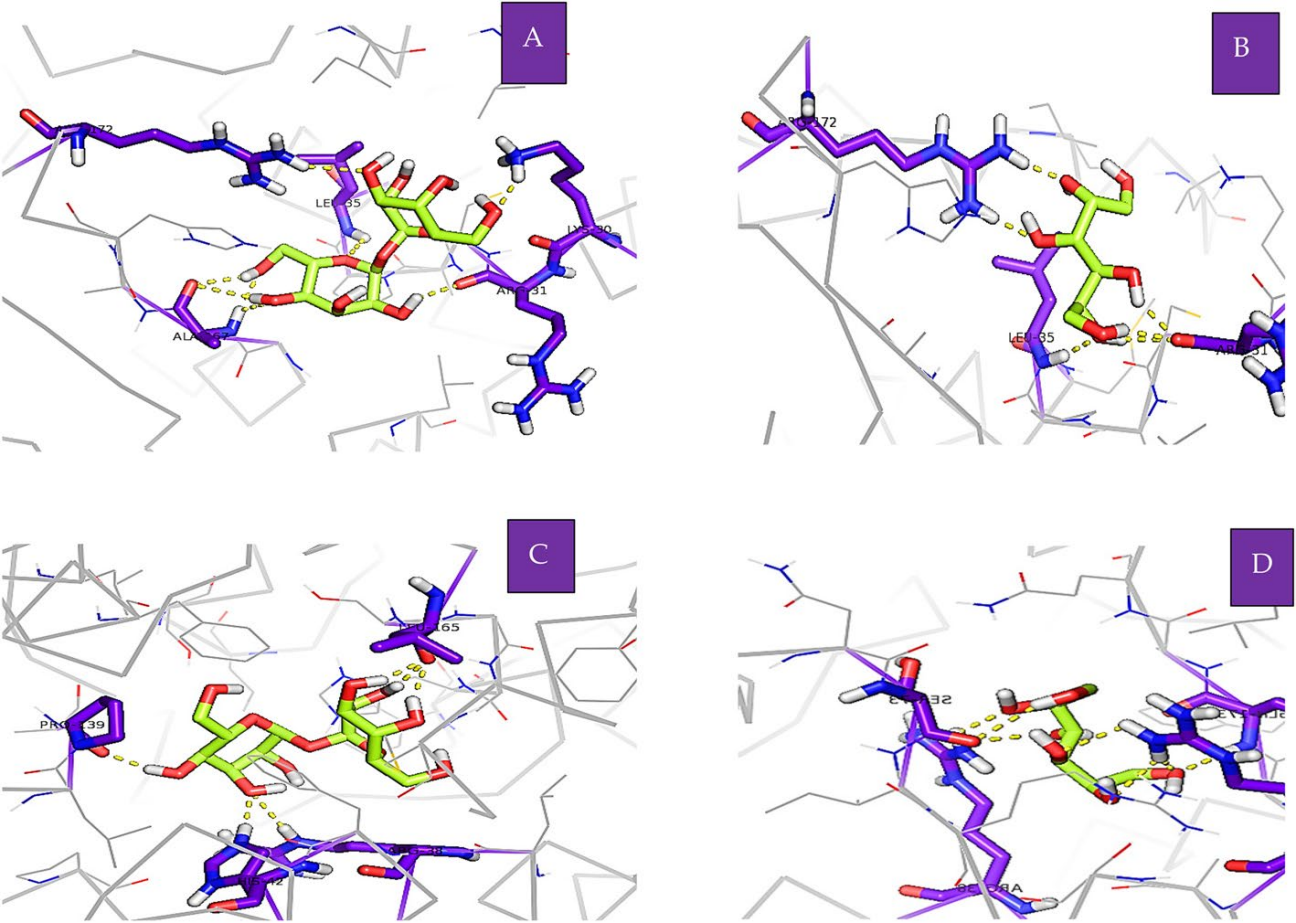
Interaction between ligands; **A** trehalose with APX, **B** mannitol with APX, **C** trehalose with G-POX, **D** mannitol with G-POX, Residues with polar interaction with ligands in purple, ligands in green and hydrogen bonds in yellow dashes.

Mannitol-treatment resulted in a significant elevation in guaiacol peroxidase activity of non-stressed wheat seedlings compared to salt-stressed seedlings. Trehalose treatment caused significantly increased in APX activity compared to control under salinity stress (Fig. 4B). To more understanding of the effect of Trehalose and mannitol on the antioxidant enzymes APX, POX and PPO molecular docking was performed. The docking was performed between the ascorbate binding site in ascorbate peroxidase 1oaf Cys32, Lys30 and Arg172, and Trehalose and mannitol. The output of the docking (Table 1) showed that both compounds bind to ascorbate binding site in the enzyme with high affinity. Trehalose binds to two residues Lys30 and Arg172 (Fig. 5A) while mannitol binds to only one residue Arg172 (Fig. 5B). Docking of Trehalose and mannitol into the active site of peroxidase 1sch Arg38, Phe41, His42, Leu138, Pro139, Ala140, Pro141, Phe142, Phe143, Leu236, Pro69, Ala71, Gln175, Thr177 and Ala178 (Table 2) showed that Trehalose binds to two residues in the binding site of hydrogen peroxide Arg38 and His42 and one residue in the substrate access channel Pro139 (Fig. 4C) while mannitol binds to only one residue in the binding site of hydrogen peroxide Arg38 (Fig. 4D).

**Table 1:** Interaction between Trehalose and mannitol and the binding site of ascorbate peroxidase

Compound	Binding Energy	Hydrogen bonds	Residues
**Trehalose**	**-3.64 kcal/mol**	**8**	**Lys30, Arg172, Leu35, Arg31 and Ala 167**
**Mannitol**	**-2.24 kcal/mol**	**6**	**Arg172, Leu35 and Arg31**

**Table 2:** Interaction between Trehalose and mannitol and the binding site of peroxidase

Compound	Binding energy	Hydrogen bonds	Residues
**Trehalose**	**-4.17 kcal/mol**	**6**	**Arg38, His42, Pro139 and Leu 165**
**Mannitol**	**-2.85 kcal/mol**	**7**	**Arg38, Arg31, Ser73 and Gln173**

**Fig. 6:**
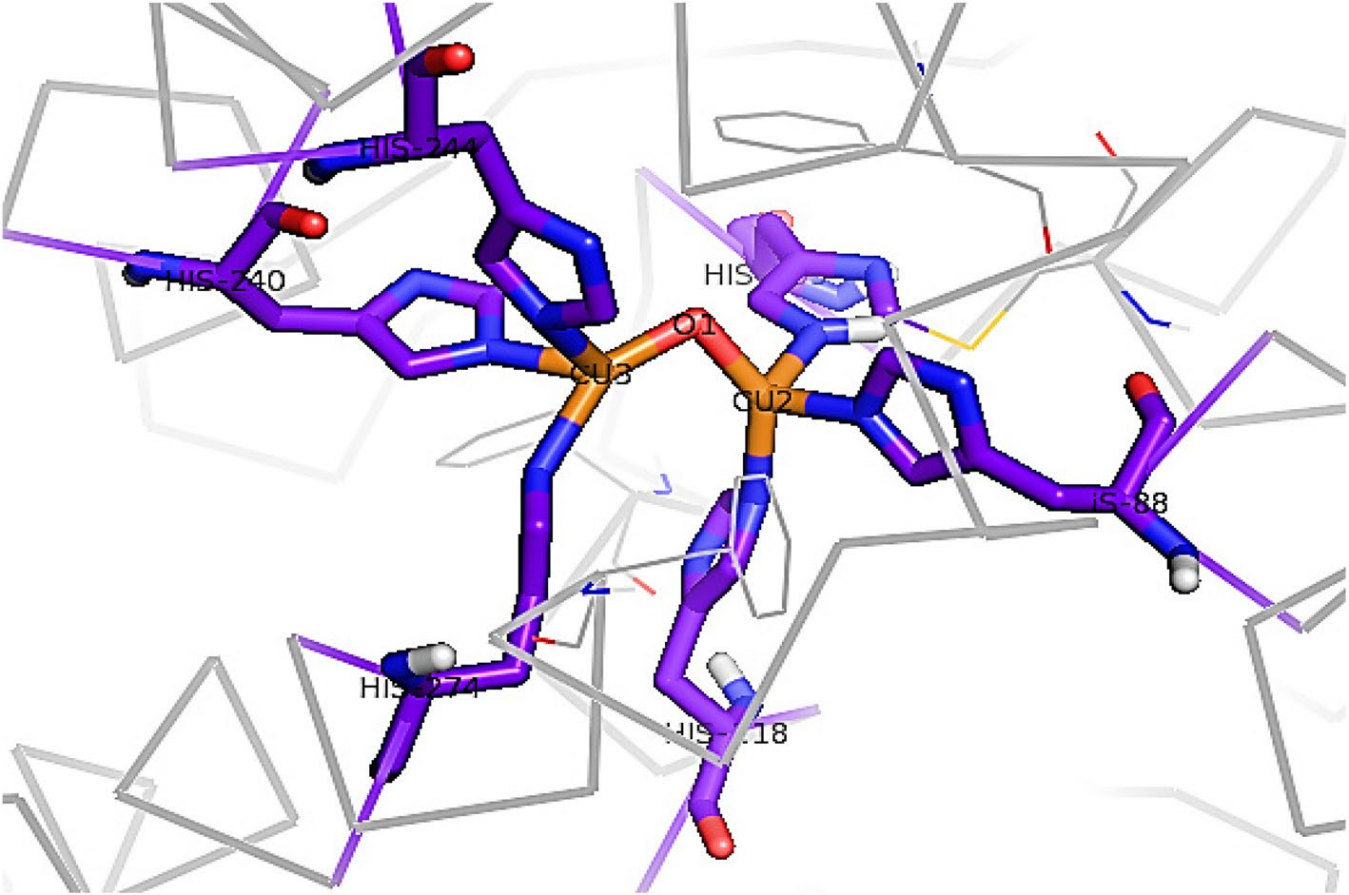
Catalytic site of PPO, residues in purple, Cu atoms in orange and oxygen atoms in red

Docking of trehalose and mannitol and polyphenol oxidase 1bt1 was performed by docking of both compounds into the hydrophobic pocket that lead the substrate to the catalytic site of PPO. The docking output showed positive binding energy which means that the both compounds have low affinity to bind to the selected site in the enzyme. Docking of Trehalose and mannitol into the catalytic site of PPO couldn’t be performed due to the Cu_2_O in the core of the catalytic site of the enzyme (Fig. 6).

### Phenylalanine ammonia-lyase activity (PAL)

**Fig. 7:**
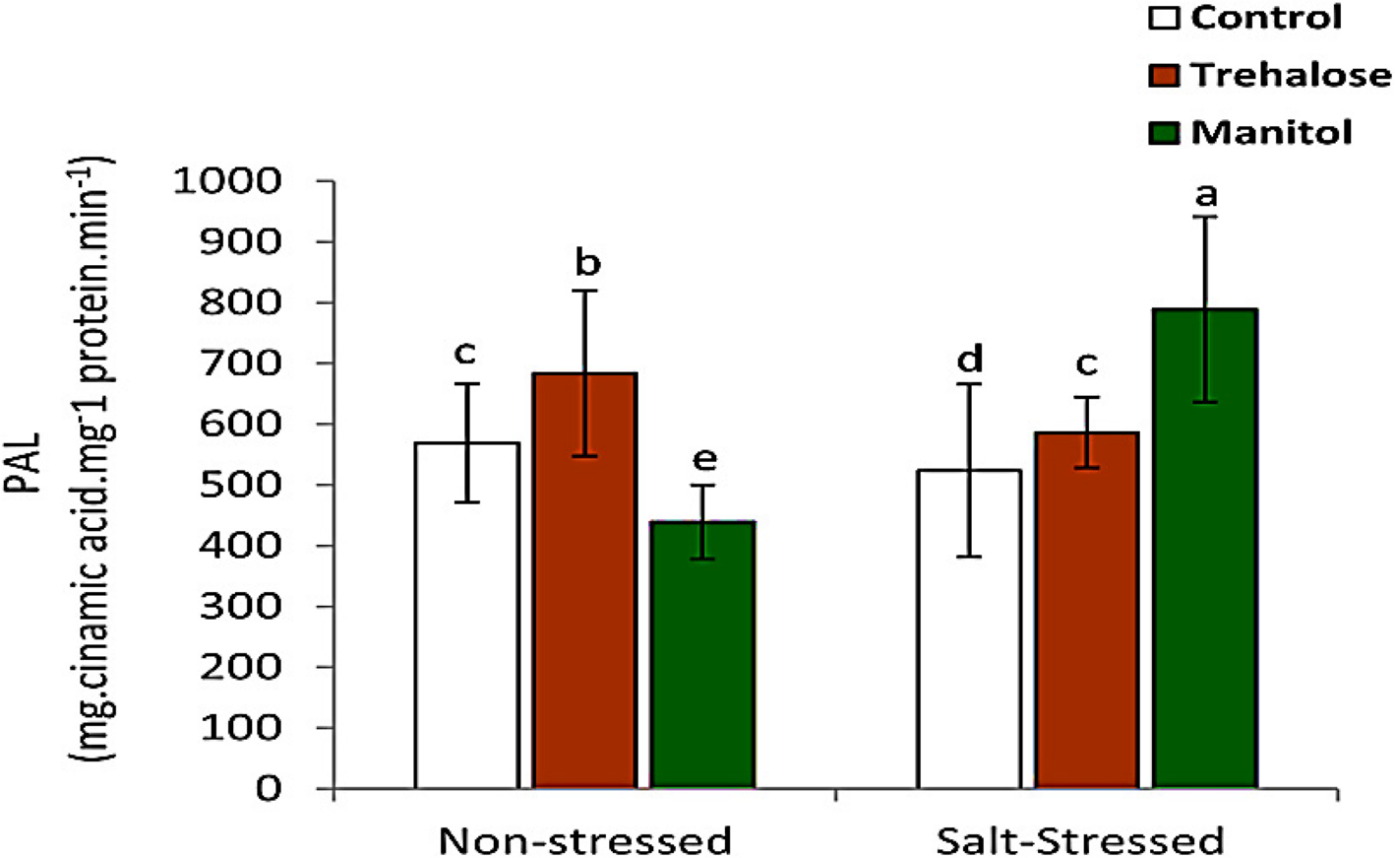
Effect of wheat grain pre-soaking in 10 mM trehalose and mannitol on Phenylalanine ammonia layase activity in wheat seedlings grown under salt stress condition.

Pre soaking wheat grains in Trehalose and mannitol treatments caused an increase in PAL activity in *Triticum estivium* seedlings grown in salt stress conditions compared to control. Also, The pre-soaking wheat grains in 10 mM trehalose resulted in a higher activity of PAL in non-stressed plants. Also, pre-soaking wheat grains in 10 mM mannitol caused a higher activity of PAL in salt-stressed wheat seedlings (Fig. 7).

## Discussion

Wheat is one of the most important crops and subjected to salinity stress in several places worldwide. Salinity stress greatly affect plant physiological processes and growth which subsequently resulted in a remarkable reduction in the productivity [37, 38]. Salt stress lead to ion toxicity, which subsequently caused osmotic and oxidative stress [39]. Therefore, to reduce the harmful effects of salt stress, plants have many defense strategies, such as scavenging reactive oxygen species (ROS) through various enzymatic and non-enzymatic antioxidants, accumulate free amino acids, proline, phenolic compounds, total and reducing sugars could stabilize cell membranes [40].

Presoaking wheat grains with Trehalose and mannitol (10 mM) elevate total soluble sugars, total phenols, total flavonoids and soluble proteins content, while free amino acids and MDA were decreased by treatments in seedlings grown under salt stress (150mM NaCl) (Figs. 1, 2 and 3). These observations are in agreement with earlier studies that used the transgenic tobacco [41, 42] and wheat [43]. Among these compatible solutes trehalose and mannitol which play an important role in plant response to abiotic stress are recently gaining attention of many researchers [44, 45]. Many researchers reported that Trehalose has a high ability to improve the tolerance of several organisms against abiotic stress. In wheat seedlings trehalose and mannitol could directly scavenge ROS including O_2_^-^ and H_2_O_2_ [46]. These results may be due to trehalose and mannitol able to improve the tolerance of wheat plant against salinity stress ,where the increase in total soluble sugars and free amino acids especially proline may be for adjusting osmotic potential and improve water uptake under salinity. These mechanism help plant to continue its growth and development under salt stress [47, 48]. Trehalose and mannitol treatments caused decrease in free amino acids while protein content was increased in seedlings under salt stress may be due to trehalose and mannitol increase conversion of free amino acids to protein for stabilize biological Structure [49]. Increase in phenolic and flavonoid compounds by trehalose and mannitol presoaking treatments which is accompanied by decrease in MDA levels due to that trehalose and mannitol are a signaling molecules encourage plants to increase biosynthesis of non enzymatic antioxidants such as phenolic compounds and flavonoids for scavenging ROS to reduce harmful effects of salt stress. So too antioxidant enzyme use phenolic compounds as a substrates in scavenging of reactive oxygen species (ROS) [50]. Phenolic compounds and flavenoids are capable of reducing lipid membrane peroxidation by scavenging ROS as well as protects plants from oxidative stress and sequently reduce MDA formation [51].

Figure 4, clearly showed that both pre soaking wheat grains with treatments Trehalose and mannitol had no significant effects on the peroxidase activity, while elevated APX and CAT activities, whereas reduced the activity of PPO in salt stressed seedlings. These results were in a harmony with Kaya et al. [52] and Ibrahim and Abdellatif [43] on *Zea mayes* and wheat respectively. Increased CAT and APX activity by as a result of treatment with trehalose and mannitol to eliminate photorespiratory H_2_O_2_ that is formed during stress [43, 53]. Trehalose acts as a signaling molecule under abiotic stresses, where it sends a signal to activate enzymatic antioxidants for scavenge ROS in order to reduce oxidative stress [54]. The molecular docking of Trehalose and mannitol prove that both molecules have direct molecular interaction with POX and APX as shown in Fig. 5. Table 1 showed that both Trehalose and mannitol have a direct interaction with ascorbate peroxidase 4 through their ability to form 8 and 6 hydrogen bonds with a negative values of energy of formation which could be a reasonable explanation of the effect of Trehalose and mannitol on the ascorbate peroxidase 4 activity. In addition, the molecular docking of Trehalose and mannitol with a peroxidase 1 indicated also a direct molecular interaction between them and the binding site of peroxidase 1 by formation of 6 and 7 hydrogen bonds respectively. The binding energy of Trehalose and mannitol to the binding site of APX is negative value of -3.64 K Cal/mole and − 2.24 K Cal / mole respectively. The molecular interaction presented in Fig. 5 suggested that the ability of Trehalose and mannitol to activate APX activity in wheat seedlings grown under salt stress condition could be resulted from their direct interaction with the enzyme. The antioxidant enzymes (CAT, POX, and APX) activity was markedly increased under Cd stress in mung bean and trehalose application increased the antioxidant enzyme activities. Therefore, Rehman et al. [55] concluded that Trehalose acts directly as signaling molecule and induced the synthesis of several metabolites and/ or indirectly improves accumulations of osmoregulatory compounds which enhance antioxidant defensive system and mitigate Cd-induced toxic effects.

Phenylalanine ammonia lyase (PAL) is a key enzyme in phenyl- propanoid pathway,which is responsible for synthesis of phenolic compounds [56]. Figure 7 Pre soaking wheat grains in Trehalose and mannitol caused an increase in PAL activity in *Triticum estivium* seedlings grown in salt stress conditions compared to control. These increase in PAL activity led to increase biosynthesis of phenolic compounds and flavonoids which scavenged ROS to reduce harmful effects of salt stress [57].

The current study clearly showed that treatment of wheat grains with 10 mM of Trehalose or mannitol induce plant tolerance against salt stress through different mechanisms including. Activation of antioxidant system, molecular interaction with antioxidant defense enzyme, activation of biosynthesis of phenols and flavenoids which acts as antioxidant.

## Conclusions

**Fig. 8:**
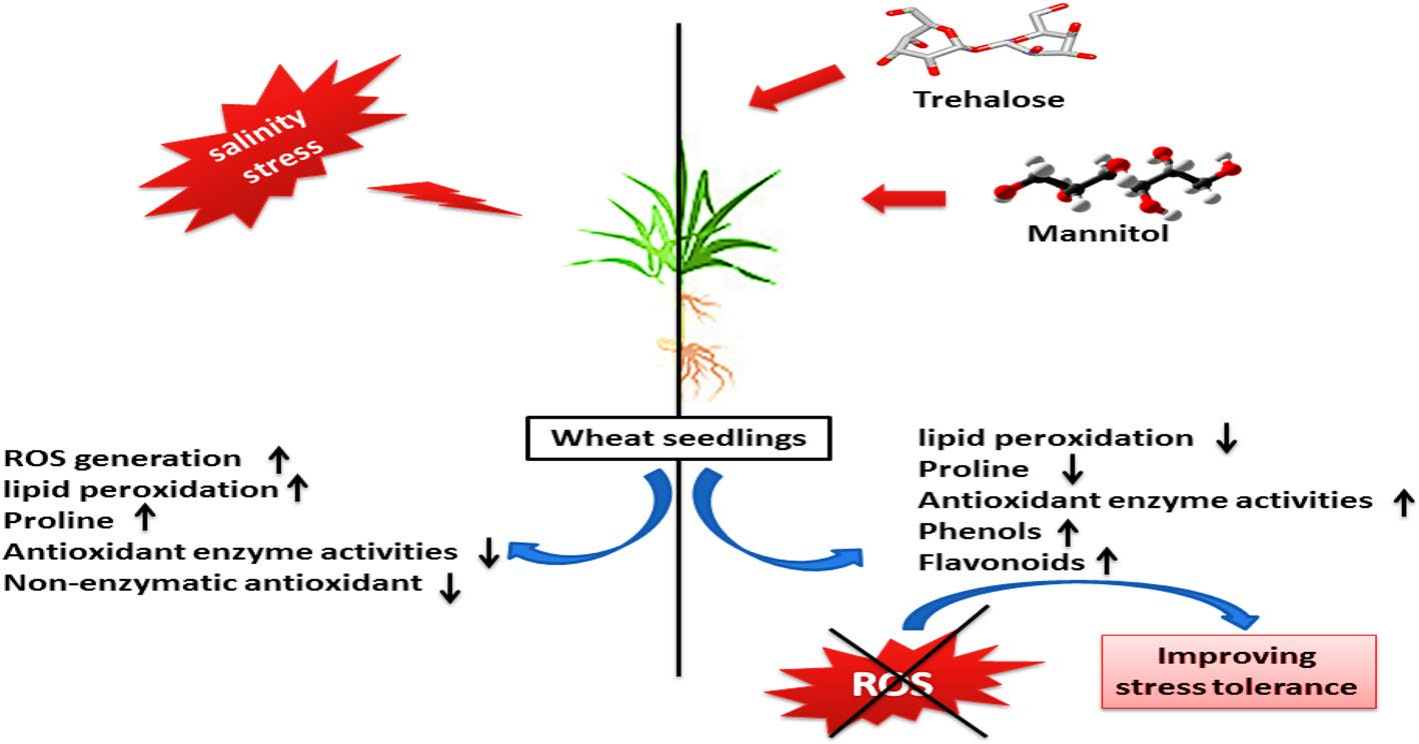
Illustration diagram summarized the effects of Trehalose and mannitol on salt- stress tolerance in wheat seedlings by mitigating the negative effects of ROS through changes of its composition from some metabolites.

The results of this study concluded that salt stress causes greater ROS formation and lipid peroxidation in wheat seedlings. The use of Trehalose and mannitol improved stress tolerance in seedlings by mitigating the negative effects of ROS. The results concluded that salt stress conditions restricted the antioxidant defense system in wheat seedlings by reducing antioxidant enzyme activities and the contents of non-enzymatic antioxidant. Wheat seedlings treated with Trehalose and mannitol before planting have improved antioxidant defence mechanisms. In this investigation, we found that Trehalose performed better than mannitol, which could be attributed to Trehalose's dual role of directly interfering with plant systems after Trehalose. In the light of preceding results, it may be concluded that Trehalose can reduce and overcome the oxidative injury through reduction of lipid peroxidation, increasing of osmoregulatory compounds such as amino acids especially proline, reducing sugars and total soluble sugars and increasing the enzymatic and non enzymatic antioxidant in wheat seedlings to maintain the balance between pro-oxidants and antioxidants like phenols and flavonoids (Fig. 8)

## Data Availability

The datasets used and analyzed during the current study are available from the corresponding author upon reasonable request.
